# Measuring pico-Newton Forces with Lipid Anchors as Force Sensors in Molecular Dynamics Simulations

**DOI:** 10.1021/acs.jpcb.3c00063

**Published:** 2023-04-26

**Authors:** Batuhan Kav, Thomas R. Weikl, Emanuel Schneck

**Affiliations:** † Max Planck Institute of Colloids and Interfaces, 14467, Potsdam, Germany; ‡ Institute of Biological Information Processing: Structural Biochemistry (IBI-7), Forschungszentrum Jülich, 52428 Jülich, Germany; § Institute for Condensed Matter Physics, Technische Universität Darmstadt, 64289 Darmstadt, Germany

## Abstract

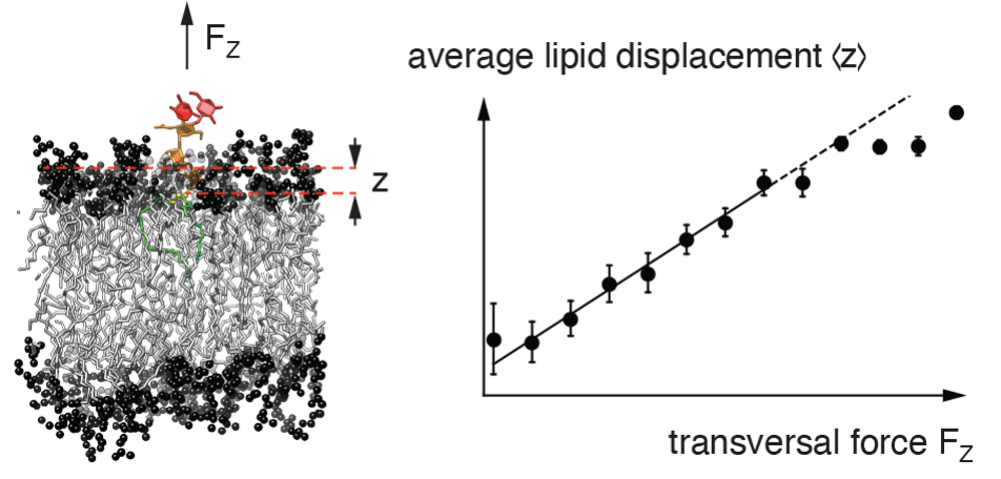

Binding forces between biomolecules are ubiquitous in nature but sometimes as weak as a few pico-Newtons (pN). In many cases, the binding partners are attached to biomembranes with the help of a lipid anchor. One important example are glycolipids that promote membrane adhesion through weak carbohydrate–carbohydrate binding between adjacent membranes. Here, we use molecular dynamics (MD) simulations to quantify the forces generated by bonds involving membrane-anchored molecules. We introduce a method in which the protrusion of the lipid anchors from the membrane acts as the force sensor. Our results with two different glycolipids reveal binding forces of up to 20 pN and corroborate the recent notion that carbohydrate–carbohydrate interactions are generic rather than specific.

## Introduction

Molecular binding forces on the scale of pN play numerous roles in biology as they can trigger cellular responses such as proliferation,^1^ migration,^2^ differentiation,^3^ tumor progression,^4^ and tissue formation.^5^ Of special interest are binding forces that can promote the adhesion of adjacent membranes. In cell adhesion, for instance, the binding partners are usually membrane proteins like cadherins, integrins, or others.^6,7^ But also membrane–anchored carbohydrates can facilitate membrane adhesion by binding to other carbohydrates present on membrane surfaces.^8−11^ The adhesion strength depends on the strength of the individual bonds and on their area density.^12−14^

The need to quantify weak biomolecular binding forces has prompted the development of experimental force sensors, for example based on magnetic or optical tweezers,^15−18^ micropipetting,^10,11,19^ or atomic force microscopy (AFM).^9,20−25^ These techniques can measure binding forces in the range of a few to few hundreds of pN. More recent studies are aimed at combining AFM with resonant energy transfer fluorescence (FRET).^26^ A recent study on T-cell receptors utilized FRET to quantify the forces that the cells exert on individual force sensor constructs.^27^

The complexity of intermolecular interactions, however, has remained a limitation to our insights into the detailed mechanisms responsible for the forces observed experimentally, which is why molecular modeling is gaining importance. Molecular dynamics (MD) simulations have been widely used to study biomolecular binding.^28^ In so-called steered MD simulations, an external force is applied along an assumed reaction coordinate,^29−31^ such that rupture forces and free energy differences can be probed.^32−38^ However, such approaches provide only limited insights into the forces exerted by the transient formation of biomolecular bonds on their environment under nonbiased equilibrium settings.^39,40^

Here, we present a computational method to determine the forces associated with bonds involving membrane molecules. It is generally applicable to all binding scenarios that exert transversal forces to lipid-anchored molecules in a range up to about 20 pN and is therefore ideally suited to investigate the role of transient weak bonds in the context of membrane adhesion. The method relies on the determination of the “spring parameters” corresponding to the lipid-anchoring potential in the direction perpendicular to the membrane plane. This can be achieved in two equivalent ways, either by Boltzmann-inversion of the equilibrium fluctuations or by the systematic application of defined transversal forces, which yield consistent results in the relevant linear force–extension regime of small out-of-plane deviations of lipid anchors. The focus on transversal forces perpendicular to the membrane is a natural consequence of the fluidity of lipid membranes, which leads to the relaxation of tangential forces in the direction of the membrane plane.^27^

Our results for lipid-anchored oligosaccharides in the form of two glycolipid types with the same lipid anchor but different sugar head groups yield rather similar force constants, suggesting that the key determining factor for the spring parameters is the anchor chemistry. With a suitable definition of bound states between sugar headgroups on the surfaces of two adjacent membrane surfaces^41^ and by averaging over hundreds of binding and unbinding events, we calculate the binding-induced protrusion of the lipid anchors and, with that, the average transversal forces, which can reach up to ≈20 pN. In fact, these force values are in good agreement with earlier reports based on AFM experiments^9^ and found to be generic rather than specific, in line with other studies.^41,42^

## Methods

### Constant–Force Pulling Simulations

Our molecular dynamics (MD) pulling simulations were carried out with version 2018.6 of the GROMACS package,^43−45^ with a 2 fs integration time step. Initial configurations and molecular topology files were converted into the GROMACS format using Acpype.^46^ In the simulations, the saccharide headgroups of the glycolipids (Le^X^ or Lac2 lipid) are described with the GLYCAM06_OSMOr14_^TIP5P^ force field,^47^ which has been optimized for use with the TIP5P water model^48^ to correct an overestimation of attractive saccharide–saccharide interactions in standard force fields.^47,49^ The matrix lipid POPC and the lipid anchors of the glycolipids are described with the AMBER Lipid14 force field^50^ with a modification^41^ that allows for its use in combination with TIP5P water. Long-range electrostatic interactions are treated with the Particle–Mesh–Ewald (PME) method^51,52^ with a real-space cutoff of 1.0 nm. Lennard–Jones interactions are cut off at 1.0 nm. Hydrogen-containing bond lengths are constrained using the SETTLE algorithm.^53^ Periodic boundary conditions (PBC) are applied in all directions. The temperature is kept at 303 K using the stochastic-dynamics (SD) integrator with a friction coefficient of τ_*t*_ = 0.5 ps. The pressure is kept at 1 bar using the standard semi-isotropic pressure coupling for membrane simulations and the Berendsen barostat^54^ with a 1 ps relaxation time.

**Figure 1:**
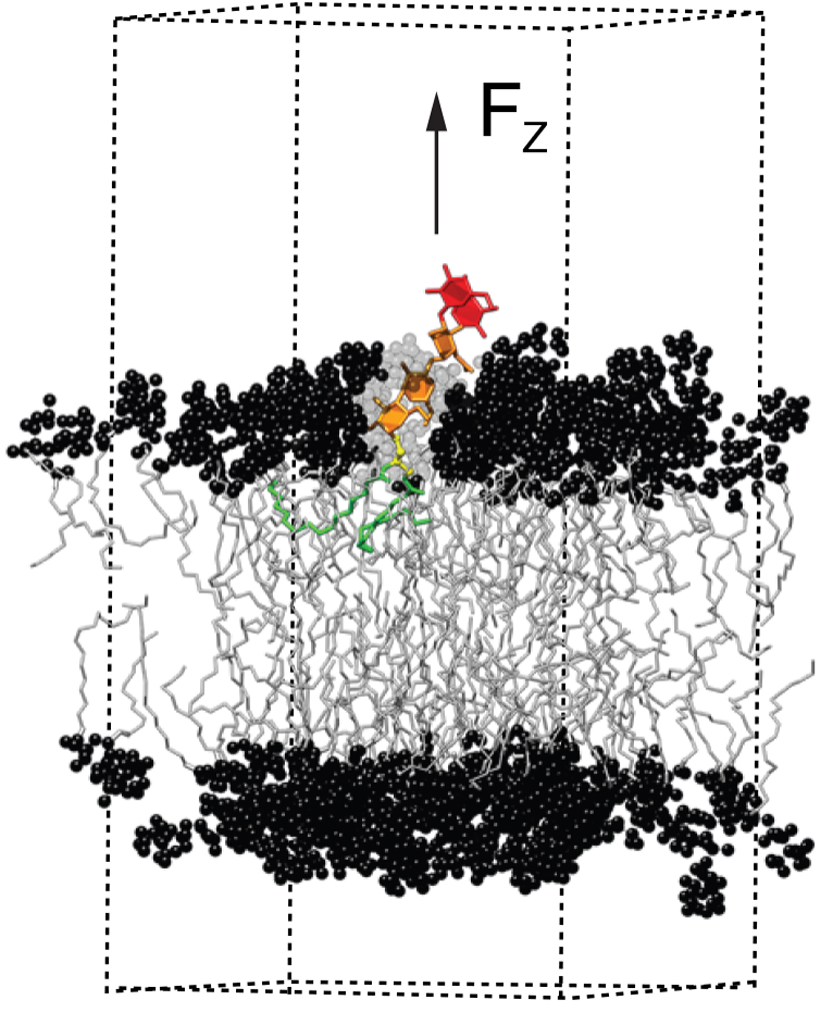
Setup for the nonequilibrium pulling simulations of the Le^X^ glycolipids. A constant force in the *z*-direction is applied between the center of mass coordinates of the glycolipid saccharide head, including linker, and the lipid bilayer. The dashed lines illustrate the simulation box. For the sake of clarity, the box height is not drawn to scale. The fucose and galactose at the branched tip of the Le^X^ glycolipids are shown in red, the linker in yellow, while the remaining parts of the Le^X^ saccharide and glycolipid tails are represented in orange and green, respectively. The lipid head groups are shown in black, and the tails are in gray. The lipid headgroup atoms around the Le^X^ glycolipid are represented as transparent to make the Le^X^ glycolipid visible.

**Figure 2:**
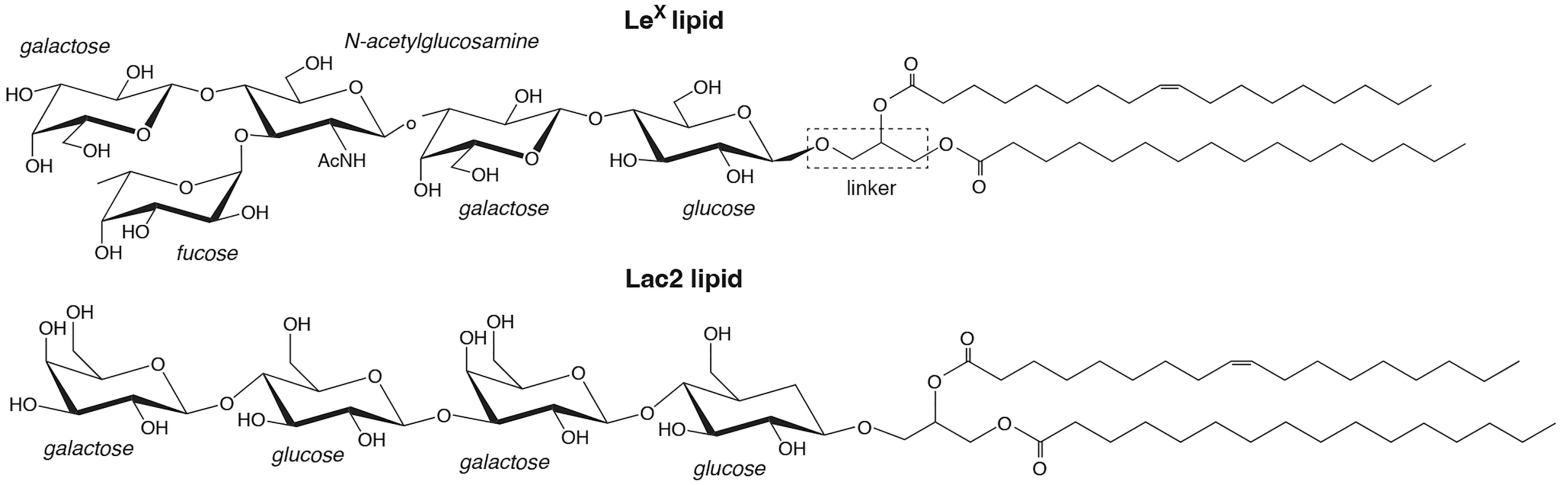
Structures of the glycolipids investigated.

The setup employed for the constant-force pulling simulations is illustrated in Figure 1. It consists of a water-immersed POPC bilayer membrane hosting a single Le^X^ lipid (see Figure 2) in one of its two monolayers, achieved by replacing one of the 36 POPC lipids per monolayer with the Le^X^ lipid, which has the same lipid tails as POPC. The box height is 25 nm in the *z* direction, and the box dimensions in the *x* and *y* directions are approximately 4.8 nm × 4.8 nm, as follows from an equilibrated average area per lipid molecule of 0.64 nm^2^ under the given conditions.^55^ A constant pulling force perpendicular to the membrane surface (i.e., in the +*z* direction) is exerted onto the glycolipid with GROMACS’ “pull–code”.^43^ This is realized by applying a constant force between the center of mass coordinates of the lipid bilayer and the glycolipid headgroup. The magnitude of the force is constant in each simulation run and systematically varied from 2.5 to 30.0 pN, with 2.5 pN increments. In order to obtain sufficient statistics, 30 independent 500 ns long trajectories are generated for each magnitude. In order to ensure full equilibration, the initial 250 ns of each trajectory are discarded and not considered in the analysis. In addition to these constant-force pulling simulations, 10 additional 1 μs long simulation trajectories of the same system without an external force are generated to obtain zero-force reference data.

### Simulations of Intermembrane Glycolipid Binding

**Figure 3:**
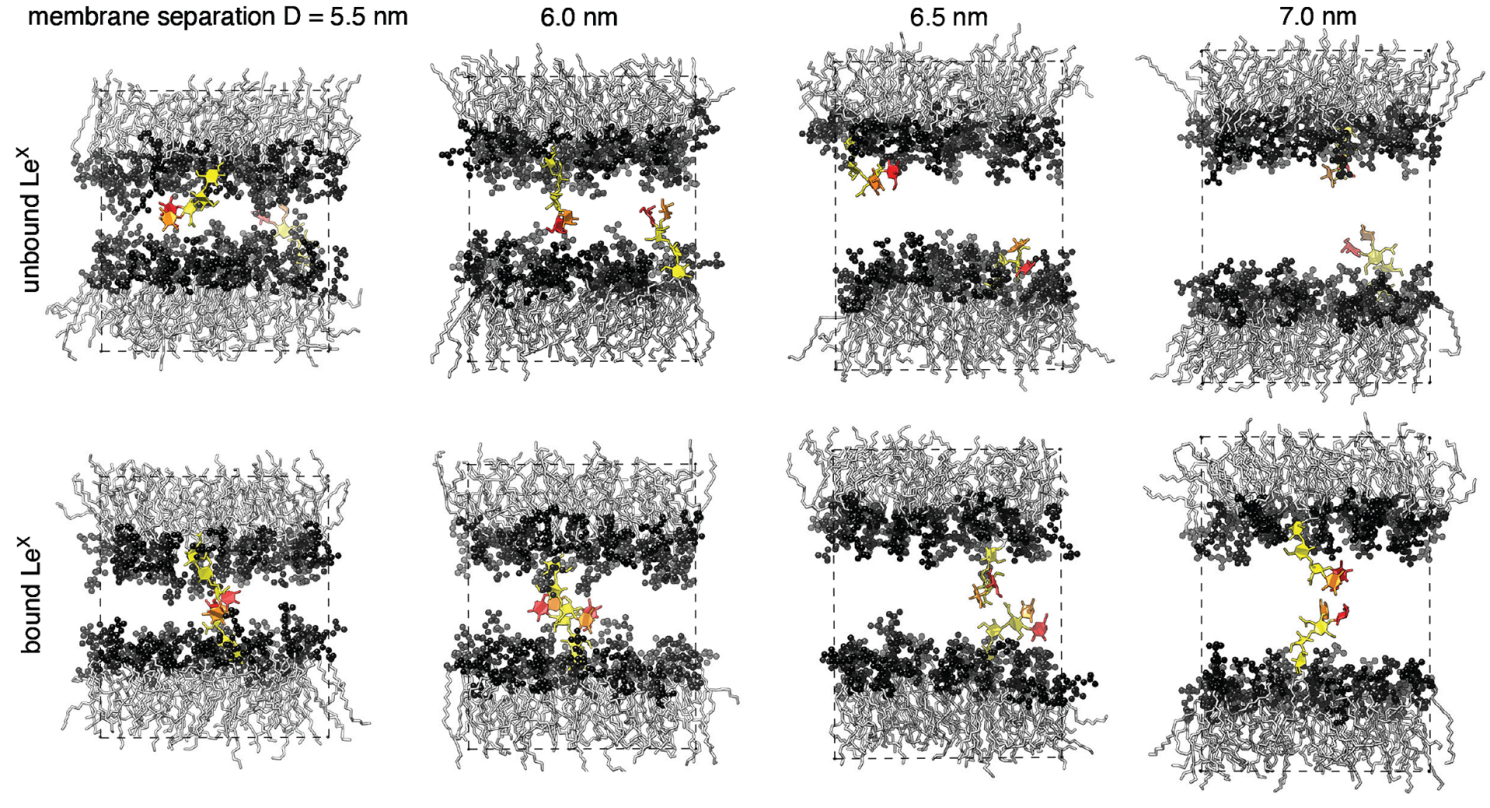
Membrane conformations from simulations to investigate the intermembrane (*trans*) binding of glycolipid headgroups. In these simulations, the membrane is composed of one glycolipid and 35 lipids in each monolayer. The two glycolipids in the different monolayers interact because of the periodic boundaries of the simulation box. The periodic boundary in vertical direction here is aligned with the membrane midplane to visualize representative unbound and bound states of two Le^X^ glycolipids at membrane separations *D* from 5.5 to 7.0 nm. The fucose and galactose at the branched tip of the Le^X^ glycolipids are represented in red and orange, and the remaining three monosaccharide units in yellow. Reproduced with permission from ref (41) under a CC-BY 3.0 license. Copyright 2020 Royal Society of Chemistry.

The setup employed to investigate the intermembrane (*trans*) binding of glycolipid headgroups is illustrated in Figure 3. In this setup, each of the two monolayers of the membrane hosts one glycolipid (Le^X^ lipid or Lac2 lipid, see Figure 2), which interacts with the glycolipid in the other monolayer across the periodic boundary of the simulation box. The separation *D* between the membrane and its periodic image, measured from center to center, can be systematically varied through variation of the height of the simulation box, or in other words, through a variation of the water layer thickness between the membrane and its periodic image. The headgroups of the two glycolipids are thus allowed to interact with each other when the membrane separation is small enough. We varied the membrane separation *D* from 5.5 to 8.0 nm in steps of 0.5 nm and performed, for each membrane separation, 10 independent simulation runs of 3 or 1 μs duration for Le^X^ lipid and Lac2 lipid, respectively, with the software AMBER 16 GPU.^56^ As in our previous studies,^41,57^*trans*-bound states are defined as time intervals (i.e., consecutive frames) with nonzero contacts between the nonhydrogen saccharide atoms, where the maximum number of contacts within the time interval is at least 5. We consider two atoms to be in contact if the distance between them is smaller than 0.45 nm.

## Results

Figure 2 shows the chemical structures of the two glycolipids, Le^X^ and Lac2 lipids. Both glycolipids were previously found experimentally to promote membrane adhesion through preferential interactions between their saccharide headgroups.^14,57^ Our previous MD simulations further revealed that the adhesion-stabilizing *trans*-bonds across the water layer are weak and fuzzy.^41^

In the following, we aim at quantifying the transversal forces (i.e., in the direction perpendicular to the membrane plane) that these *trans*-bonds are subject to in the context of membrane adhesion. For this purpose, we monitor the force-induced protrusions of the *trans*-bonded glycolipids and subsequently deduce the magnitude of the associated transversal forces. The protrusion δ*z* = *z* – *z*_0_ of a glycolipid is the instantaneous displacement of the linker region (defined as the vertical center of mass coordinate *z* of the atom group indicated in Figure 2) from its average position *z*_0_ in the absence of any force. The absolute reference point *z* = 0 is the instantaneous ensemble-averaged center of mass position of the POPC headgroups that belong to the same bilayer leaflet. The slightly negative value of *z*_0_ (≈−0.3 nm, see Table 1) reflects that the linker atoms are situated a bit deeper in the membrane than the headgroup atoms of the matrix lipids.

**Table 1:** Spring Constant *k* and Force-Free Reference Position *z*_0_ of the Linker Region

	unbiased MD sim.^41^		constant force pulling sim.	
	*k* (pN/nm)	*z*_0_ (nm)	*k* (pN/nm)	*z*_0_ (nm)
Le^X^	94 ± 4	–0.31 ± 0.01	86 ± 5	–0.34 ± 0.01
Lac2	109 ± 2	–0.31 ± 0.06		

In the first step, we establish the relation between the transversal force *F*_*z*_ and the time-averaged protrusion of a glycolipid
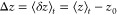
1with the help of constant-force pulling simulations (see Figure 1), in which a defined artificial transversal force is exerted to the saccharide headgroup of the glycolipids (see Methods). Subsequently, we test to what extent this relation can be obtained more economically also via Boltzmann inversion of the force-free distribution of equilibrium protrusions. In the second step, we exploit the known relation *F*_*z*_(Δ*z*) to determine the average transversal forces acting on *trans*-engaged glycolipids involved in the adhesion of adjacent membranes as a function of the membrane separation *D*.

### Force–Protrusion Relation from Constant-Force Pulling Simulations

**Figure 4:**
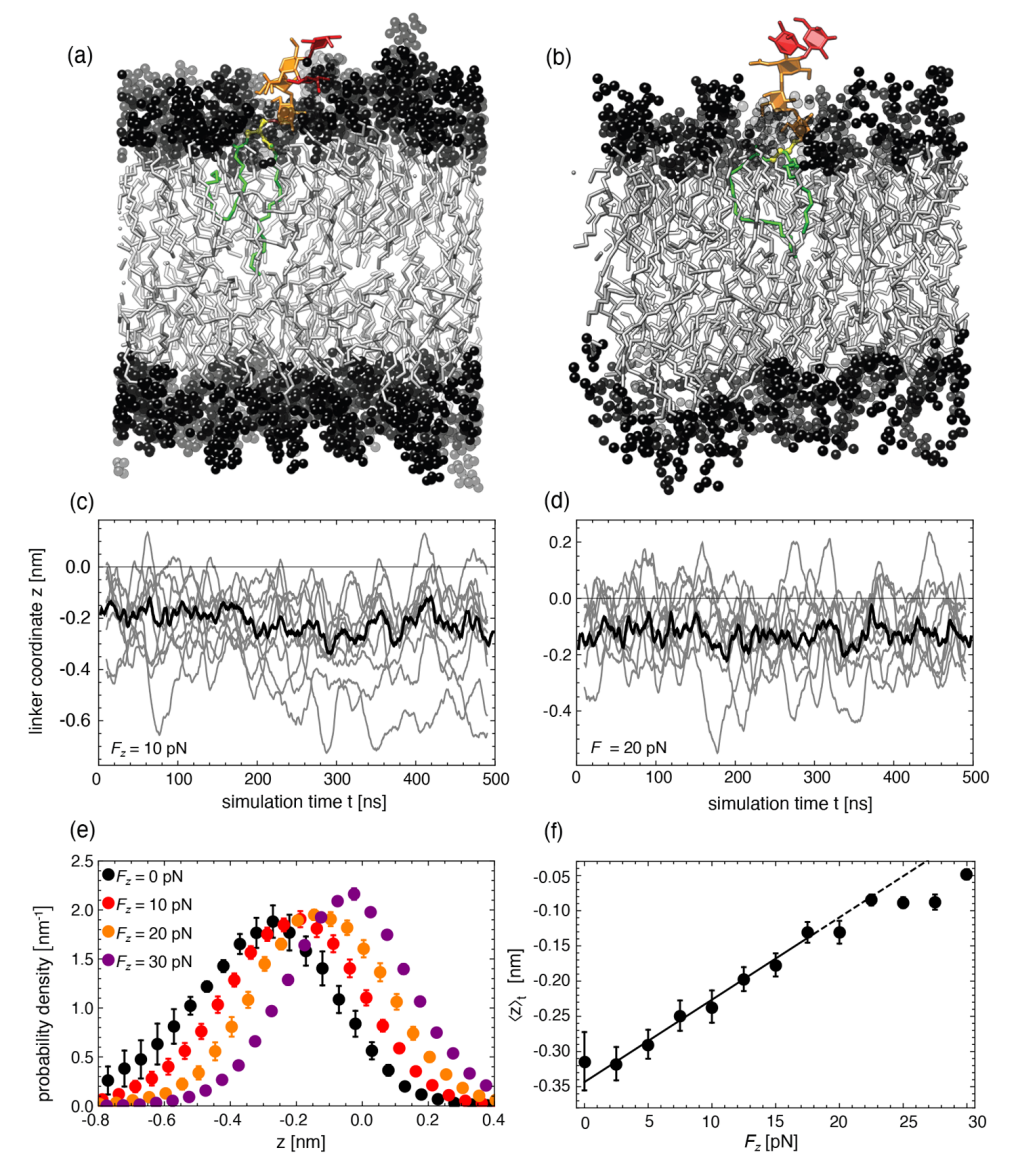
(a, b) Snapshots from constant force pulling simulations with a Le^*X*^ lipid for *F*_*z*_ = 0 pN (a) and *F*_*z*_ = 30 pN (b). The Le^X^ glycolipids and lipids are represented as in Figure 1. (c, d) Linker coordinate *z* as a function of simulation time at the transversal forces *F*_*z*_ = 10 and 20 pN, along 10 randomly chosen trajectories (gray lines, smoothened over 20 ns, i.e., over 200 frames at intervals of 0.1 ns) and as a time-dependent average over all 30 trajectories (black lines, smoothened over 5 ns). (e) Force-dependent probability densities of the *z*-position of the linker. For clarity, data are only shown for selected forces, *F*_*z*_ = 0, 10, 20, and 30 pN. The data points represent averages and standard errors over 30 independent runs. (f) The resulting force–extension curve. The black line indicates an error-weighted linear fit to the data points between 0 and 20 pN. The dashed line is a linear extrapolation beyond the fitting range.

Figure 4 shows snapshots from a constant-force pulling simulation with a single Le^X^ lipid in a POPC bilayer. The force-free reference is shown in panel (a). In panel (b), the saccharide headgroup of the glycolipid experiences a constant transversal pulling force of *F*_*z*_ = 30 pN. The force not only brings the headgroup into a stretched configuration but also displaces the linker region more to the membrane periphery. Panels (c) and (d) illustrate the relaxation and equilibration of the vertical linker coordinate *z* along simulation trajectories at the forces *F*_*z*_ = 10 and 20 pN, respectively. Panel (e) shows the associated distributions of *z* for selected values of *F*_*z*_, featuring a systematic shift to higher *z* values with increasing force. Panel (f) shows the distributions’ center of mass (COM) position, ⟨*z*⟩_*t*_, as a function of *F*_*z*_. It is seen that ⟨*z*⟩_*t*_ increases virtually linearly with *F*_*z*_ up to *F*_*z*_ ≈ 20 pN, like for a Hookean spring. The solid straight line indicates a linear fit to the data points with *F*_*z*_ ≤ 20 pN. Its intercept defines *z*_0_ and, with that, the average displacement Δ*z* = ⟨*z*⟩_*t*_ – *z*_0_ (see eq 1). The slope encodes the spring constant, *k*, which approximates the *F*_*z*_(Δ*z*) curve within a harmonic model. For Le^X^, we obtain *z*_0_ = −0.34 ± 0.01 nm and *k* = 86 ± 5 pN/nm from the linear fit (see Table 1 for an overview), with error margins indicating standard errors of the fit. At higher forces, a sublinear behavior is observed, corresponding to anharmonic hardening of the spring. For the highest forces applied here (*F*_*z*_ = 30 pN), the average protrusion reaches Δ*z* ≈ 0.3 nm.

**Figure 5:**
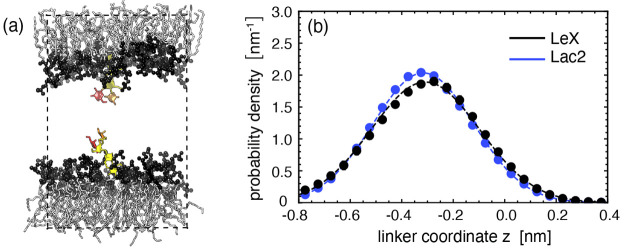
(a) Snapshot from a simulation of two Le^X^ lipids in an essentially planar membrane at a large separation (here: *D* = 8.0 nm), where the saccharide headgroups are geometrically unable to engage in a *trans*-bond. The bottom leaflet is translated by the height of the simulation box for visual purposes. (b) Corresponding force-free distributions of *z* for Le^X^ lipid and Lac2 lipid as obtained from this type of simulation.

Next, we aim at extracting the relation *F*_*z*_(Δ*z*) alternatively from the equilibrium distribution of the instantaneous protrusions in the absence of an external force. Figure 5a shows a simulation snapshot of a membrane containing one unbound Le^X^ lipid in each leaflet. The membrane separation is as high as *D* = 8.0 nm, so that the saccharide headgroups are geometrically unable to engage in a *trans*-bond. Panel (b) shows the corresponding force-free distributions of *z* for Le^X^ lipids and Lac2 lipids as obtained from this type of simulation. In the following, the bell-like shape of the distribution is again described with a harmonic spring model. The associated potential energy *V* has the form
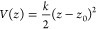
2In thermal equilibrium, the probability is Boltzmann-distributed
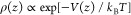
3where *k*_B_ and *T* are the Boltzmann constant and the temperature, respectively. For a harmonic potential, this distribution has the shape of a Gaussian function,
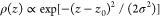
4with the standard deviation
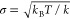
5The spring constant *k* can thus be extracted from a Gaussian fit to the distribution ρ(*z*) according to eq 4. With *z*_0_ and *k* at hand, the harmonic potential (eq 2) is fully defined, and we can calculate the associated force
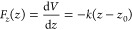
6which, when averaged over time (see eq 1), yields the sought-after force–protrusion relation
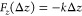
7The values of *z*_0_ and *k* obtained from Gaussian fitting for the Le^X^ and Lac2 lipids are summarized in Table 1, with error margins representing standard errors of fit values obtained from the 10 independent trajectories. It is seen that the spring constants obtained via Boltzmann inversion and via constant force pulling are consistent within their error margins, which represent standard errors. The harmonic approximation (eq 2) in the present case seems to hold up to forces of about 20 pN. In this regime, calibration curves from pulling simulations are not necessary and can be replaced by computationally more economical unbiased simulations, as was done in our earlier study.^41^ Nevertheless, a calibration curve obtained by constant–force pulling must be considered the most robust approach, especially when higher forces are at play. Noteworthy, the calibration curves obtained with the two lipid species are very similar (see Table 1), confirming the intuitive expectation that what matters is the lipid anchoring. It can therefore be anticipated that *k* as well as the extension of the harmonic regime are sensitive to the chemical details (such as length and saturation) of the tails of the anchoring lipid, but also of the matrix lipids.

### Transversal Forces on *trans*-Engaged Glycolipids

**Figure 6:**
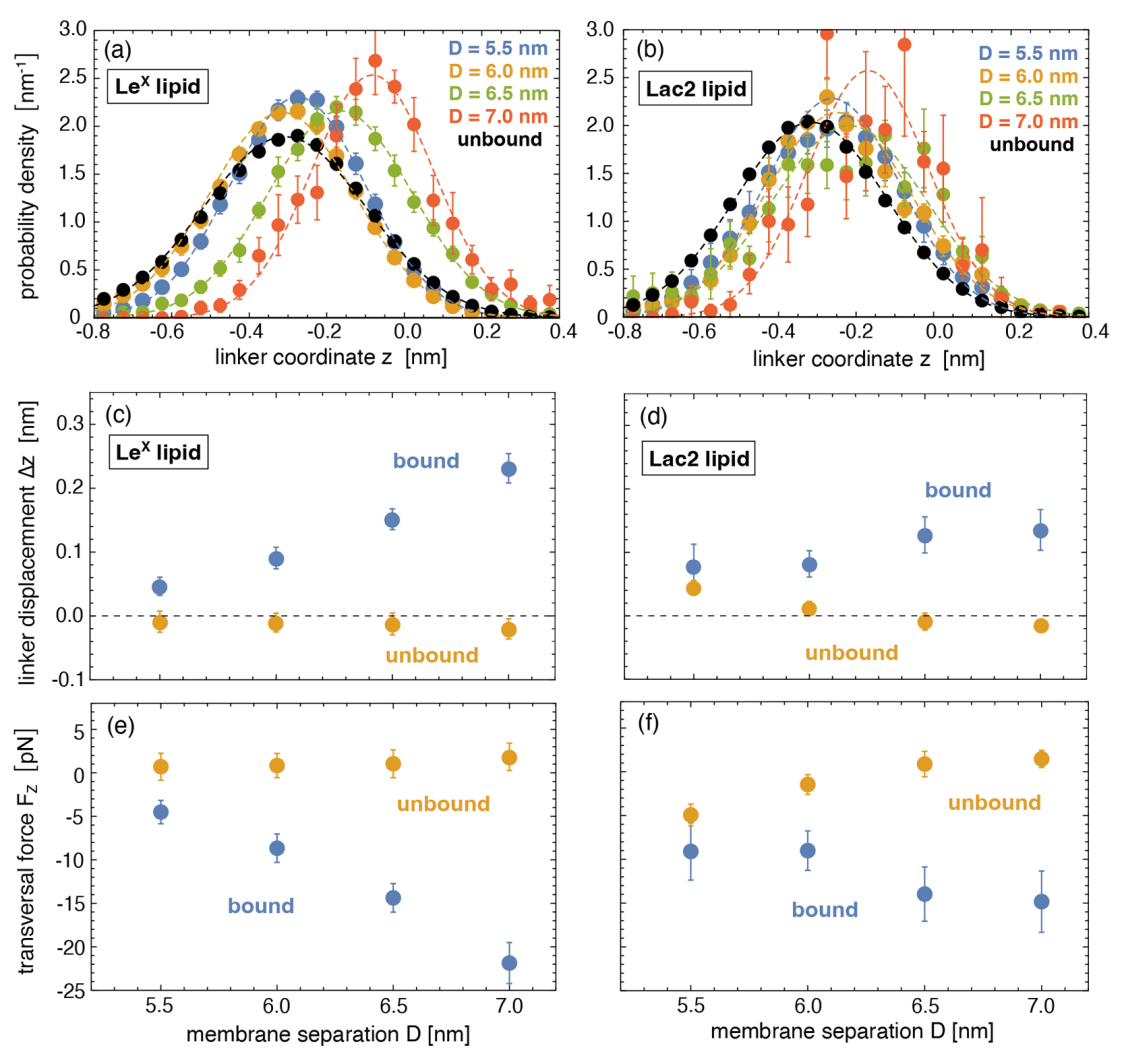
(a, b) Distributions of vertical linker coordinate *z* associated with the *trans*-engaged (bound) states of Le^X^ lipids (b) and Lac2 lipids (c) for various membrane separations *D* (see also Figure 3), together with the reference distributions of the unbound states for *D* = 8.0 nm. (c, d) Time-averaged protrusions Δ*z* of bound and unbound states as functions of the membrane separation *D* for Le^X^ lipids and Lac2 lipids. (e, f) Associated transversal forces *F*_*z*_ according to eq 7 for Le^X^ lipids (e) and Lac2 lipids (f).

Now we turn to scenarios in which two glycolipids belonging to the opposing membrane surfaces are able to get engaged in intermembrane (trans) binding. As detailed in the Methods section, this is realized by systematically reducing the membrane separation *D*, as illustrated in Figure 3. Figure 6a,b shows the *z*-distributions of *trans*-engaged (“bound”) Le^X^ and Lac2 lipids at various membrane separations *D*. For reference, the respective distributions for the unbound glycolipids in noninteracting membranes are included in these plots in black color. The definition of a bond is provided in our earlier work^41^ and in the Methods section. In order to get engaged in a *trans*-bond, the glycolipids have to protrude more or less from the membrane surface, depending on *D*. This tendency is reflected in the distributions in Figure 6a,b, which exhibit systematic shifts to less negative *z* when *D* increases. On the other hand, increasing *D* also reduces the probability for the glycolipids to be bound, which leads to poorer statistics in the associated distributions (see error bars in Figure 6a,b). This reduced binding probability is also reflected by a strong decrease in the binding constants with increasing separation *D*.^41^

Figure 6c,d shows the time-averaged protrusions Δ*z* of bound glycolipids according to eq 1 for Le^X^ lipids and Lac2 lipids as a function of the membrane separation *D*. It is seen that Δ*z* increases monotonically with *D* for Le^X^ lipids. However, for Lac2 lipids, Δ*z* rather seems to saturate after an initial increase. This saturation suggests that *trans*-binding with saccharide configurations of different out-of-plane extensions (as imposed by different membrane separations) can be associated with similar displacements of the linker region. Equation 7 allows us to convert Δ*z*(*D*) into the average transversal force *F*_*z*_ exerted on the trans-engaged glycolipids. Figure 6e,f shows *F*_*z*_ as a function of the membrane separation. A gradual increase in the membrane separation up to *D* = 7 nm leads to a systematic increase in the force up to *F*_*z*_ = 23 ± 3 pN for Le^X^ lipids and *F*_*z*_ = 15 ± 3 pN for Lac2 lipids. For larger membrane separations, the occurrence of *trans*-engaged glycolipids becomes negligible. The obtained maximum force values are in agreement with unbinding forces of 20 ± 4 pN per Le^X^ bond reported in an experimental work that used atomic force microscopy measurements.^9^ To the best of our knowledge, no experimental data on the Lac2 lipid are available. However, the similar values of the maximum binding forces observed for the Le^X^ and Lac2 lipids suggest that the preferential interactions between the saccharide headgroups are generic rather than chemistry-specific, in line with recent experimental findings.^42^

For the two glycolipid types investigated, the forces acting on the headgroup-anchoring point are found to be always tensile (= negative). What is remarkable is that this is the case even at smaller membrane separations, where steric confinement likely occurs and positive forces would be naively expected. A possible explanation for this behavior could be that glycolipid protrusions out of the bilayer generally facilitate trans-engaging, which leads to some shift in the distributions in the positive *z*-direction. Figure 6c,d show Δ*z*(*D*) also for the unbound glycolipids, as a control. For Le^X^ lipids, the approach of the opposing membrane surface does not lead to any measurable protrusion of the unbound molecules, which is in line with the expectation. Interestingly, for Lac2 lipids, Δ*z* exhibits a tiny yet apparently significant shift to positive values for the two smallest membrane separations investigated (*D* ≤ 6.0 nm). While we do not have any definite explanation for this behavior, one possible reason could be a “lever effect”, where the anchoring part of the lipid gets slightly lifted out of the membrane when the comparatively long linear tetrasaccharide headgroup of Lac2 lipids interacts sterically with the surface of the opposing membrane. Why this effect should be less pronounced for Le^X^ lipids is, however, not clear at the moment.

## Discussion

The spring parameters obtained for Le^X^ and Lac2 lipids are rather similar, indicating that they are governed by the lipid anchoring alone. Therefore, our method could be extendable, in principle, to any lipid-anchored molecule, such as lipid–DNA force sensors,^58,59^ Ras proteins,^60,61^ or GPI anchors.^62−65^ For a given combination of membrane composition and lipid anchor, the spring parameters need to be calibrated only once. Our method provides two possible ways to obtain the spring parameters. The first way is to run unbiased MD simulations of the lipid-anchored molecules without any binding candidates and to determine the *k* and *z*_0_ by application of eqs 4 and 5. The second way is to employ constant-force pulling simulations and to extract *k* and *z*_0_ according to eq 7. In the linear force–extension regime of small out-of-plane deviations of lipid anchors, these two ways yield results that are identical within standard errrors (see Table 1). The force regime considered here is distinct from simulation regimes aiming at extracting lipids out of a membrane via umbrella-sampling^66,67^ or pulling simulations with constant pull rate,^29^ which lead to much larger out-of-plane deviations of lipids and to significantly larger transversal forces.

The data points in Figures 5 and 6 are based on 10 independent trajectories with duration of 3 μs (Le^X^ lipid) or 1 μs (Lac2 lipid). The relative errors associated with the obtained spring properties are on the order of few %. Therefore, already shorter simulations would likely be sufficient to determine spring properties with satisfactory precision. However, we would like to emphasize that μs-simulations of membranes and membrane-anchored molecules are becoming routinely accessible thanks to modern hardware and software.^56,68−72^ To generate the data points in Figure 4, we performed 30 independent simulations of 500 ns per force value. These simulations, similar to the unbiased MD simulations, are computationally easily tractable. For the average *trans*-deviation for a given force value, the magnitude of the relative errors is correlated inversely with the applied force. Therefore, it is also possible to run fewer/shorter simulations for the larger force values. Using the constant-force simulations, it is also easy to verify the range of forces that can be faithfully estimated from our model. From a practical point of view, unbiased MD and constant–force pulling simulations provide equivalent results.

In our simulations, the lipid bilayers are essentially planar due to the small membrane sizes and, in the case of unbiased MD simulations, also because the glycolipid in one monolayer interacts with the glycolipid in the other monolayer of the membrane across the periodic boundary of the simulation box. In simulations with larger membranes, the membranes undergo shape fluctuations,^57^ which requires an adjustment of the quantification of out-of-plane deviations by considering, e.g., a lipid disk of a few nanometers around a lipid anchor as reference, rather than the whole membrane. In the case of heterogeneous bilayer compositions, it would be important to use membrane disks that are large enough to have overall the same compositions as the local interactions between the different lipids and the lipid-anchored molecule can affect the anchoring potential.^29^

## Conclusions

We have introduced a computational method to quantify weak binding forces mediated by the preferential interaction of glycolipids in opposing membrane surfaces. The protrusion of the lipid anchors from the membrane acts as force sensor. Two independent methods for the calibration of the force versus protrusion relation yield consistent results. Depending on the membrane separation, the maximum binding forces observed between the glycolipids bearing Le^X^ and Lac2 headgroups were of the order of 20 pN and therefore much weaker than those of typically probed biomolecular binding partners. Our method appears to be generally applicable to measuring pN forces with lipid anchors as force sensors in molecular dynamics simulations.
